# Standardized *Salvia miltiorrhiza* Extract Suppresses Hepatic Stellate Cell Activation and Attenuates Steatohepatitis Induced by a Methionine-Choline Deficient Diet in Mice

**DOI:** 10.3390/molecules19068189

**Published:** 2014-06-17

**Authors:** Hak Sung Lee, Woo-Chan Son, Jae-Eun Ryu, Bon Am Koo, Yeong Shik Kim

**Affiliations:** 1 Natural Products Research Institute, College of Pharmacy, Seoul National University, 1 Gwanak-ro, Gwanak-gu, Seoul 151-742, Korea; E-Mail: mildpeople@snu.ac.kr; 2 Research Center, Samil Pharmaceutical Co. Ltd., 216 Sandan-ro, Danwon-gu, Ansan 425-852, Korea; 3 Department of Pathology, University of Ulsan College of Medicine, Asan Medical Center, 88 Olympic-ro 43-gil, Songpa-gu, Seoul 138-736, Korea; E-Mails: wcson32@hanmail.net (W.-C.S.); bangarje@naver.com (J.-E.R.)

**Keywords:** *Salvia miltiorrhiza*, non-alcoholic steatohepatitis (NASH), methionine-choline deficient (MCD), hepatic stellate cell (HSC), TGF-β1, TNF-α, MMP-2, MMP-9, tanshinone IIA

## Abstract

The aim of this study was to examine the effect of standardized extract of *Salvia miltiorrhiza* (SME) on gene and protein expression of non-alcoholic steatohepatitis (NASH)-related factors in activated human hepatic stellate cells (HSC), and in mice with steatohepatitis induced by a methionine-choline deficient (MCD) diet. Male C57BL/6J mice were placed on an MCD or control diet for 8 weeks and SME (0, 0.1, 0.5 and 1 mg/kg body weight) was administered orally every other day for 4 or 6 weeks. HSCs from the LX-2 cell line were treated with transforming growth factor β-1 (TGF-β1) or TGF-β1 plus SME (0.1–10 μg/mL). To investigate the effect of SME on reactive oxygen species (ROS)-induced condition, LX-2 cells were treated with hydrogen peroxide (H_2_O_2_) or H_2_O_2_plus SME (0.1–100 μg/mL). MCD administration for 12 weeks increased mRNA expression of tumor necrosis factor (TNF-α), TGF-β1, interleukin-1β (IL-1β), C-reactive protein (CRP), α-smooth muscle actin (α-SMA), type I collagen, matrix metalloproteinase-2 (MMP-2) and MMP-9. TGF-β1-induced LX-2 cells exhibited similar gene expression patterns. SME treatment significantly reduced the mRNA and protein expression of NASH-related factors in the mouse model and HSCs. Histopathological liver analysis showed improved non-alcoholic fatty liver disease (NAFLD) activity and fibrosis score in SME-treated mice. The *in vivo* studies showed that SME had a significant effect at low doses. These results suggest that SME might be a potential therapeutic candidate for NAFLD treatment.

## 1. Introduction

Non-alcoholic fatty liver disease (NAFLD) is regarded as the most common cause of abnormal liver disease in many populations [1]. Non-alcoholic steatohepatitis (NASH) belongs to the spectrum of NAFLD, and is characterized by steatosis, inflammation and ballooning degeneration with or without fibrosis in the liver [2]. NASH is a leading cause of hepatic fibrosis and, if left untreated, can lead to progressive liver diseases such as cirrhosis and hepatocellular carcinoma [3,4,5]. Increased oxidative stress, inflammatory cytokine up-regulation and hepatocyte apoptosis are believed to play important roles in the pathogenesis of NASH [6,7,8]. Animal models of NASH have greatly contributed to the understanding of its pathogenesis and molecular mechanisms. These models include genetic models such as the leptin-deficient (ob/ob) or leptin-resistant (db/db) mouse, and dietary methionine-choline deficient (MCD) models [9]. The MCD diet impairs mitochondrial β-oxidation and leads to increased reactive oxygen species (ROS) production, mitochondrial DNA damage and apoptotic cell death, in addition to hepatic stellate cell (HSC) activation and extracellular matrix deposition [10]. Oxidative stress has been implicated as an aetiological factor in many acute and chronic liver diseases including alcoholic steatohepatitis (ASH) and NASH [11]. 

According to the past “two hits theory” for the pathogenesis of NASH, the reactive oxygen products that increase in the “second hit” cause accumulation of lipid peroxidation products, mitochondrial dysfunction, and the increased secretion of pro-inflammatory cytokines such as tumor necrosis factor-alpha (TNF-α) [12]. But recently, the pathogenesis of NASH including these second-hits is now regarded as a “multiple-parallel hit” process that multiple hits act together in the development of NASH [13,14]. TNF-α is an inflammatory cytokine that plays a major role in the progression from steatosis to NASH [7], and causes secretion of various other cytokines and chemokines. Among these secreted cytokines, the most important one is transforming growth factor-β (TGF-β), which plays a pivotal role in hepatic fibrogenesis through HSC activation [15,16]. HSC activation by TNF-α and TGF-β results in the expression and deposition of smooth muscle α-actin (α-SMA) and type-(I, IV) collagen [17]. TNF-α and interleukin-1 (IL-1) also modulate several matrix metalloproteinases (MMPs) that are involved in liver injury, repair and remodeling [18], with MMP-2 and MMP-9 the most relevant MMPs with regards to liver disease [19]. These two MMPs have similar gelatin binding domains to fibronectin, through which they bind type I or type IV collagen [20]. HSCs activated to a myofibroblast phenotype secrete MMP-2 [21]. MMP-9 is found at sites of acute wound healing and in the scar regions of active fibrosis, indicating that HSCs, as well as macrophages, may be an important source of MMP-9 [22]. 

**Figure 1:**
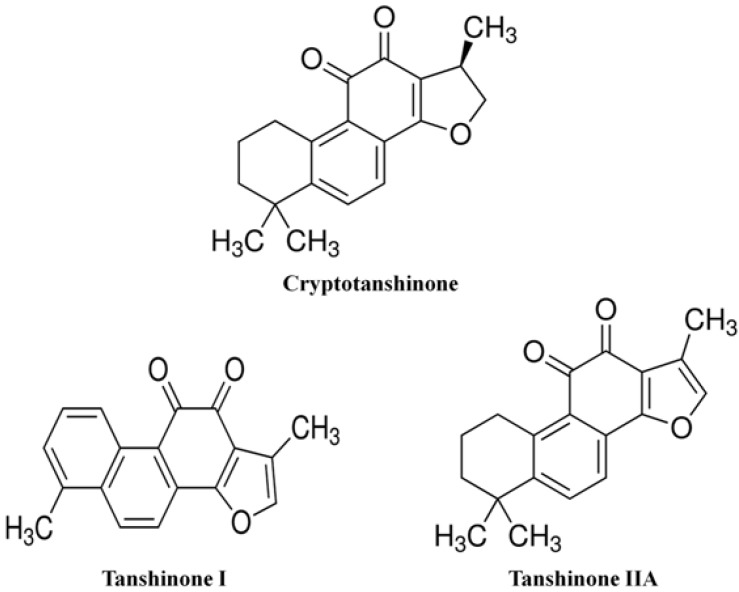
Structures of cryptotanshinone, tanshinone I and tanshinone IIA.

*Salvia miltiorrhiza* Bunge (Labiatae) is a valuable medicinal herb in Asian countries, whose dried root has been traditionally used in multiple herbal medications. The key constituents of the *S*. *miltiorrhiza* root can be classified into two major groups, a hydrophilic group of phenolic compounds such as the salvianolic acids, and a lipophilic group of diterpene quinone pigments such as the tanshinones [23]. Among the tanshinone compounds, tanshinone I, tanshinone IIA, and cryptotanshinone (Figure 1) are the major bioactive agents possessing anti-inflammatory, antioxidant and anti-tumor activities [24,25,26]. 

In particular, tanshinone IIA exhibits stronger anti-inflammatory activity than the other tanshinone derivatives. Previous studies show that tanshinone IIA is a key anti-inflammatory modulator and exerts its effects by inhibiting the activity of nitric oxide (NO), IL-1β, IL-6 and TNF-α [27]. According to Yin *et al*. reported that tanshinone IIA have potential to inhibit alcoholic liver disease by ethanol-induced cytopathogenic model [28]. Tanshinone IIA alleviated hepatocellular steatosis through oxidative stress regulation *in vitro* [29]. Tanshinone IIA can also down-regulate MMP-2 and MMP-9 activity in a high fat diet model [30]. A number of studies examining the treatment of NASH have evaluated anti-inflammatory and/or anti-fibrogenic roles of natural product-derived compounds; however, the signaling pathways activated by these compounds in the liver are still not fully understood.

The aim of the present study was to investigate the effects of the *Salvia miltiorrhiza* extract, containing 40% tanshinone IIA, in an *in vivo* model of experimental steatohepatitis and related hepatic fibrosis induced by the MCD diet and *in vitro* in human HSCs induced by TGF-β1.

## 2. Results and Discussion

### 2.1. Body Weight and Clinical Observations

The MCD diet caused a marked and progressive decrease in mouse body weight. Furthermore, irrespective of SME treatment, body weight declined slowly until death (Table 1). Three, five and four mice in the low-, mid- and high-dose SME group, respectively, exhibited yellow crusts around the mouth at 6 weeks. After microscopic examination, this clinical sign was attributed to malnutrition and starvation. Importantly, mice fed the MCD diet lost weight due to a vastly reduced caloric intake. Since most humans with NASH are obese and insulin resistant, this represents an important difference between the MCD dietary mouse model and human NASH [31]. 

**Table 1:** Body (g) and liver/body weight (%) of animals in each group before treatment (8 weeks) and after treatment (4 and 6 weeks) during the experimental protocol.

	Body Weight (g)	Liver/Body Weight (%)
***8 weeks***		
Normal diet	27.03 ± 0.5155	
MCD diet	16.76 ± 0.2428	
***12 weeks***		
Normal control	26.76 ± 0.7128	3.144 ± 0.06255
MCD	17.01 ± 0.7240	4.405 ± 0.1035
MCD+Low	16.72 ± 0.6787	3.924 ± 0.1320 *
MCD+Mid	16.63 ± 0.6992	4.277 ± 0.1320
MCD+High	16.31 ± 0.6780	3.814 ± 0.1272 **
***14 weeks***		
Normal control	27.30 ± 0.7598	3.282 ± 0.09449
MCD	16.95 ± 0.7444	3.751 ± 0.2258
MCD+Low	17.16 ± 0.6988	4.035 ± 0.06351
MCD+Mid	16.75 ± 0.7078	4.303 ± 0.3289
MCD+High	16.54 ± 0.6764	3.928 ± 0.2047

Mice were administered a methionine and choline-deficient diet (MCD) or treated with SME. At the beginning of the protocol, every other week, and at the time of killing body weight were recorded. Liver weight were recorded at the time of killing. Data are expressed as mean ± SD, * *p* < 0.05 *vs.* MCD diet only; ** *p* = 0.0070 *vs.* MCD diet only.

### 2.2. Liver Weight

The relative liver weight was higher in groups fed the MCD diet than in mice fed the control diet. The relative liver weight of mice treated with SME was significantly reduced compared to the MCD diet group at 4 weeks (*p* < 0.05). Fatty livers weigh more than normal livers. Mice fed the MCD diet had reduced body weight but increased relative liver weight, indicating that the MCD diet induced fatty liver development. The low- and high-dose SME groups had decreased relative liver weights suggestive of a reduction in fatty liver development. However, SME administration did not result in major changes to relative liver weight in all groups at 6 weeks, which may be due to an adaptation to the nutritional imbalance or low statistical power due to deaths before necropsy (Table 1).

### 2.3. Mortality

In the 4-week study, six mice died in total: three, one, and two died in the low-dose SME group, high-dose SME group, and in the control group fed the MCD diet during the administration period, respectively. In the 6-week study, 26 mice died in total: six, six, seven, and seven died in the low-, mid- and high-dose SME groups, and in the control group fed the MCD diet during the administration period, respectively. There was no notable difference in mortality between SME treatment groups and the MCD-only fed group. The cause of death was considered to be liver malfunction due to the prolonged MCD diet (see Supplementary Material). 

### 2.4. Histopathology Analysis

**Figure 2:**
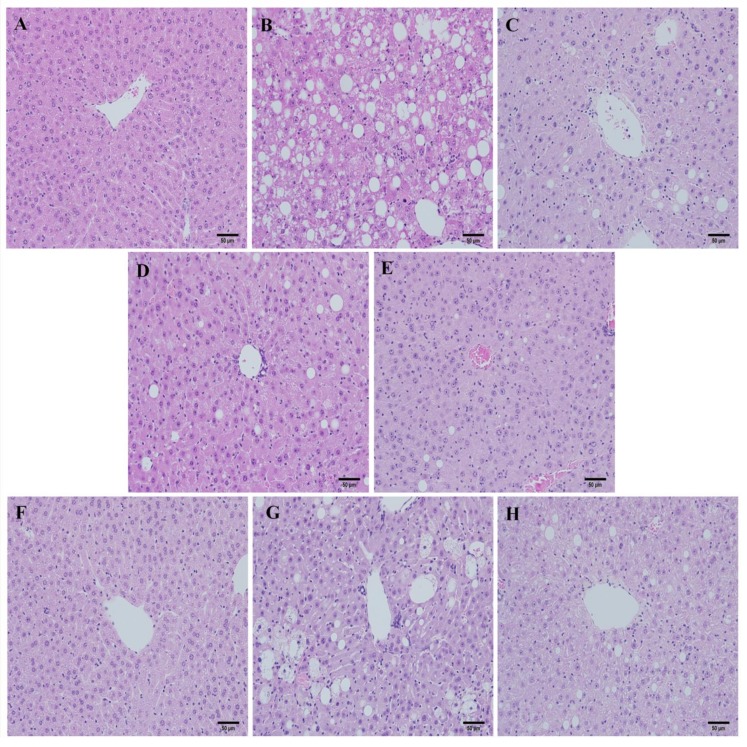

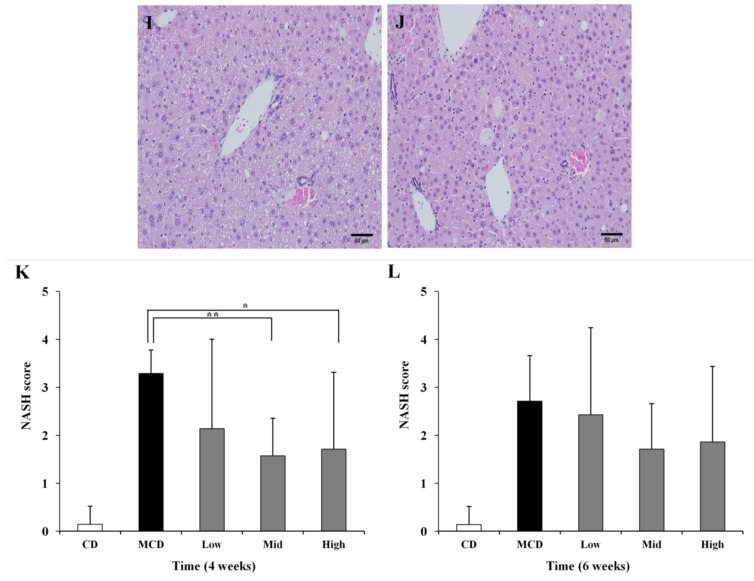
Effects of SME administration on NASH induced by the MCD diet in liver sections stained with H&E. (**A**) Normal group (normal diet only); (**B**) Control group (MCD diet only); (**C**) Low-dose group (SME; 0.1 mg/kg/day); (**D**) Mid-dose group (SME; 0.5 mg/kg/day); (**E**) High-dose group (SME; 1 mg/kg/day) for 4 weeks (magnification ×200) and (**F**) Normal group (normal diet only); (**G**) Control group (MCD diet only); (**H**) Low-dose group (SME; 0.1 mg/kg/day); (**I**) Mid-dose group (SME; 0.5 mg/kg/day); (**J**) High-dose group (SME; 1 mg/kg/day) for 6 weeks (magnification ×200); (**K**,**L**) Sections were evaluated in a blinded manner by pathologists, and a scoring method was assigned as described in experimental methods for NASH (NAFLD Activity Score) evaluation. Data are expressed as mean ± SD, * *p* < 0.05 *vs.* MCD diet only; ** *p* = 0.0004 *vs.* MCD diet.

Liver sections from the normal group stained with hematoxylin and eosin (H&E) showed normal hepatic cells and histoarchitecture (Figure 2A). By contrast, sections from mice fed the MCD diet showed massive hepatocyte ballooning and steatosis, with mild inflammatory cell infiltration (Figure 2B). The MCD diet induced measurable hepatic steatosis (mainly with a predominant macrovesicular pattern) in mice and this progressed to inflammation and fibrosis. These changes were associated with hepatic cellular damage and oxidative stress. The mechanism for steatosis includes impaired very-low-density lipoprotein (VLDL) secretion due to the lack of phosphatidyl choline synthesis [32]. It is possible that the MCD diet induced hepatic triglyceride accumulation through the blockage of hepatic VLDL secretion and inhibition of mitochondrial fatty acid β-oxidation [33]. 

Liver histology was evaluated using the NASH score. Slides were evaluated in a blinded manner and scored for steatosis, ballooning, and inflammation. At 4 weeks, the NASH score of the group fed the control diet (CD) was 0.1429. By contrast, the NASH score of the MCD-fed group was 3.286. SME treatment resulted in an improvement of the liver inflammation associated with the MCD diet. Hepatic lesions, hepatocyte ballooning and fatty degeneration were significantly reduced in the mid-dose SME group (Score: 1.571) (Figure 2D and high-dose SME group (Score: 1.714) (Figure 2E) compared to the MCD-only fed group (Figure 2B). At 6 weeks, SME treatment resulted in a slight liver improvement compared to the MCD-only fed mice, but this was not statistically significant (Figure 2H–J,L). The healthy liver is first sensitized by excessive triglyceride accumulation, and subsequently exposed to inflammatory and oxidative stress, which results in the development of NASH. These data indicate that SME treatment significantly improves hepatic cellular damage, inflammation and oxidative stress in the liver of mice fed the MCD diet. 

Although steatosis and inflammation appear early during the course of experimental steatohepatitis, fibrosis is a late event that has a deep impact on prognosis, causing hepatocellular dysfunction and the emergence of portal hypertension. In normal livers, collagen is only present in the walls of major blood vessels. If fibrosis is severe, collagen fiber appears in other areas and fibrous expansion of portal areas, with marked portal-to-portal as well as portal-to-central bridging, is observed. Liver fibrosis was analyzed in mice receiving the MCD diet with the Sirius Red stain, which stains collagen fibers a dark pink color. Fibrosis in the liver appeared after six weeks of the MCD diet, surrounding the centrilobular vein and creating a fine network of fibers around groups of hepatocytes. 

**Figure 3:**
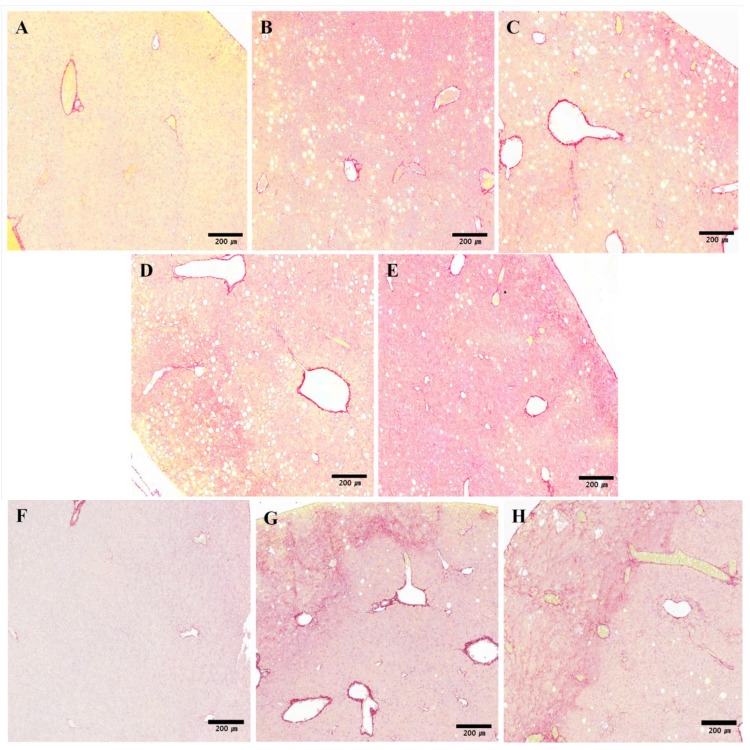

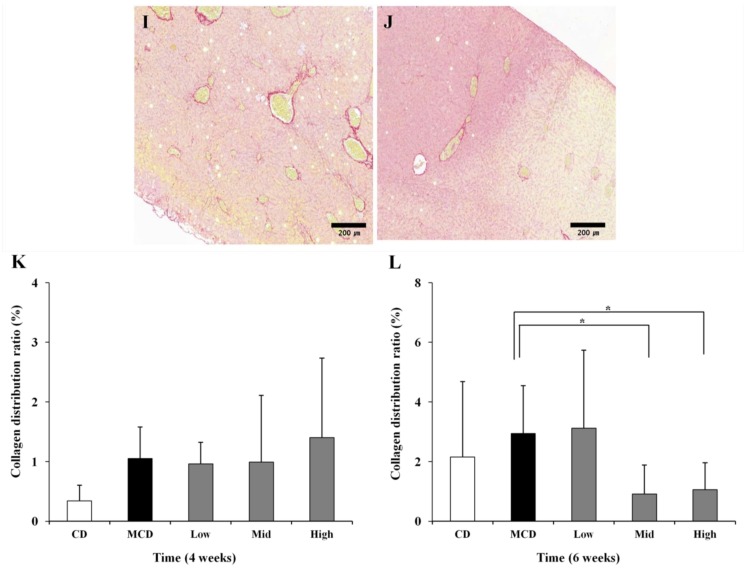
Effects of SME on histopathological changes induced by MCD diet in liver sections stained with Sirius Red. (**A**) Normal group (normal diet only); (**B**) Control group (MCD diet only); (**C**) Low-dose group (SME; 0.1 mg/kg/day); (**D**) Mid-dose group (SME; 0.5 mg/kg/day); (**E**) High-dose group (SME; 1 mg/kg/day) for 4 weeks (magnification ×200) and (**F**) Normal group (normal diet only); (**G**) Control group (MCD diet only); (**H**) Low-dose group (SME; 0.1 mg/kg/day); (**I**) Mid-dose group (SME; 0.5 mg/kg/day); (**J**) High-dose group (SME; 1 mg/kg/day) for 6 weeks (magnification ×200); (**K**,**L**) Quantification of collagen accumulation is expressed as mean ± SD, * *p* < 0.05 *vs.* MCD diet only.

At 6 weeks, livers from mice treated with SME showed a reduction in fibrosis (Figure 3H–J) compared with livers from mice fed the MCD diet only (Figure 3G). The collagen ratio in the liver parenchymal was calculated to give a quantitative measure of fibrosis. The collagen distribution ratio of the normal group was 0.34% at 4 weeks and 2.15% at 6 weeks. The distribution ratio slightly increased in all groups at 6 weeks compared to 4 weeks, suggesting a time-dependent change. There were no differences between groups at 4 weeks, though the collagen distribution increased slightly in the high-dose SME group (Figure 3A–E); however, SME administration decreased the collagen distribution ratio at 6 weeks. Although the collagen distribution ratio of the low-dose SME group (3.12%) was not significantly different compared to the control group (2.94%), the collagen distribution ratios of the mid-dose SME group (0.91%, *p* < 0.05) and high-dose SME group (1.06%, *p* < 0.05) were significantly decreased compared to the control group (Figure 3G). This result suggests that a 6-week treatment with SME significantly attenuates the development of liver fibrosis induced by the MCD diet.

### 2.5. Stability Test of Tanshinone IIA

**Figure 4:**
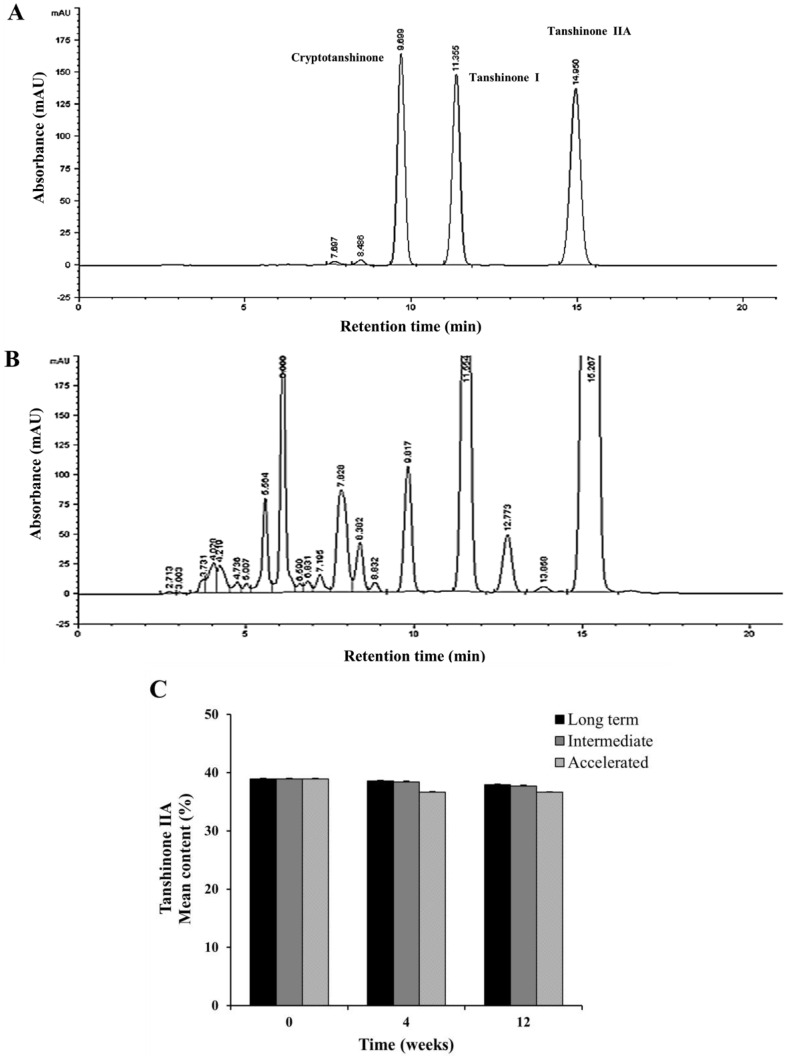
Effect of storage conditions on tanshinone IIA content in SME. (**A**)The HPLC chromatogram of the standard tanshinone mixture solution shows an absorption peak with a retention time of 14.950 min; (**B**) This peak is also present in the HPLC chromatogram of the SME, indicating the presence of tanshinone IIA; (**C**) The storage conditions did not significantly change the tanshinone IIA content between the initial test and the final test.

To investigate whether the percentage of tanshinone IIA content in SME is reduced by various storage conditions, a stability test was performed. In detail, for the accelerated, stressed and long-term stability test, SMEs were stored at 40 °C/75%, 60 °C/75% and 25 °C/60% relative humidity (RH) for 12 weeks, respectively (see Supplementary Material). The HPLC chromatograms of the standard tanshinone mixture (cryptotanshinone, tanshinone I and tanshinone IIA) solution showed an absorption peak with a retention time of 14.950 min. This peak was also present in the HPLC chromatogram of the SME, indicating the presence of tanshinone IIA (Figure 4A, B). Figure 4C shows that the different storage conditions did not significantly alter the tanshinone IIA content between the initial test (38.9% ± 0.15%) and the final test (37.9% ± 0.09%). 

### 2.6. Effect of SME on Hepatic TNF-α and Collagen I Expression in NASH Induced by the MCD Diet in Mice

**Figure 5:**
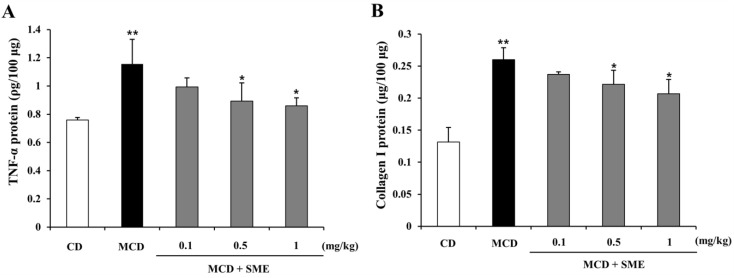
Effect of SME on hepatic TNF-α and collagen I protein levels in mice fed the MCD diet Comparison of mice fed with MCD diet only for 12 weeks (control) and treated with SME or vehicle by oral gavage from week 9 to 12. (**A**) TNF-α protein level. Values are mean ± SD (*n =* 5–7 per group), ** *p* < 0.01 *vs.* control diet; * *p* < 0.05 MCD diet *vs.* SME treated; (**B**) Collagen I level. Values are means ± SD (*n =* 5–7 per group), ** *p* < 0.01 *vs.* control diet; * *p* < 0.01 MCD diet *vs.* SME treated.

Figure 5 shows that with the progress to NASH, hepatic protein expression of TNF-α and collagen I in the MCD-fed group were significantly increased compared to expression in the CD group (1.52-fold for TNF-α and 1.97-fold for collagen I). Compared to mice fed with the MCD diet only for 12 weeks (control), mice treated with SME (0.1–1 mg/kg) for 4 weeks, after an 8-week MCD diet, showed lower hepatic protein expression of TNF-α (0.14 to 0.26-fold) (Figure 5A) and collagen I (0.09 to 0.20-fold) (Figure 5B). 

### 2.7. Effect of SME on Hepatic mRNA Expression of NASH-Related Specific Genes in the MCD Dietary Mouse Model

**Figure 6:**
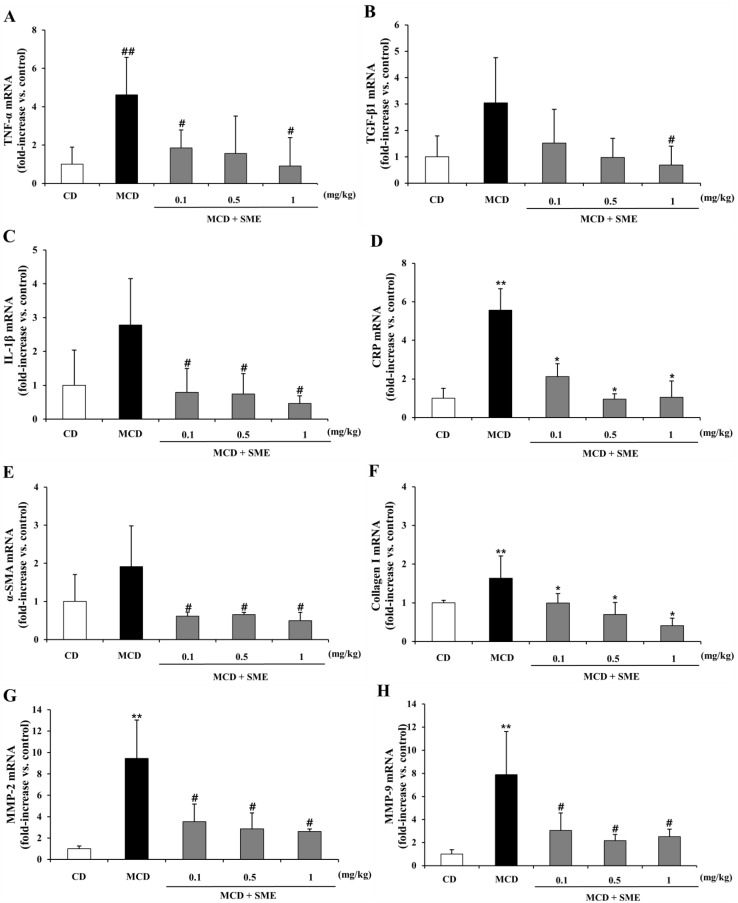
Effect of SME on hepatic mRNA expression of NASH-related specific genes in the livers of mice fed the MCD diet. Comparison of mice fed with MCD diet only for 12 weeks (control) and treated with SME or vehicle by oral gavage from week 9 to 12. (**A**) TNF-α; (**B**) TGF-β1; (**C**) IL-1β; (**D**) CRP; (**E**) α-SMA; (**F**) Collagen I; (**G**) MMP-2; (**H**) MMP-9. The mRNA expression level, normalized to GAPDH levels, is represented as fold induction of control and shown as mean ± SD (*n =* 5–7 per group), ** *p* < 0.01 *vs.* control diet; **^##^** *p* < 0.05 *vs.* control diet; * *p* < 0.01 MCD diet *vs.* SME treated; ^#^ *p* < 0.05 MCD diet *vs.* SME treated.

The MCD diet induces severe ROS production, apoptotic cell death, HSC activation and extracellular matrix deposition [10,34]. The mRNA expression levels of NASH-related genes associated with pro-inflammatory cytokines and fibrogenesis for extracellular matrix (ECM) remodeling were measured by quantitative real-time PCR analysis in the liver tissue. Figure 6A–H show that in the MCD-fed group, TNF-α, TGF-β1, IL-1β and C-reactive protein (CRP) mRNA expression levels were increased by 4.62-, 3.04-, 2.77- and 4.01-fold, respectively, compared to the CD-fed group. However, treatment with SME (0.1–1 mg/kg) for 4 weeks, after the MCD diet for eight weeks, reduced the mRNA expression of TNF-α (0.60 to 0.80-fold) (Figure 6A), TGF-β1 (0.50 to 0.77-fold) (Figure 6B), IL-1β (0.71 to 0.83-fold) (Figure 6C) and CRP (0.47 to 0.74-fold) (Figure 6D), relative to expression levels in mice fed the MCD diet only. Expression of fibrotic genes showed similar patterns. The mRNA expression of α-SMA, collagen I, MMP-2 and MMP-9 was increased by 1.91-, 1.63-, 9.44- and 7.89-fold, respectively, in the MCD diet-fed group compared to the CD-fed group. However, treatment with SME (0.1–1 mg/kg) for 4 weeks, after the MCD diet for 8 weeks, reduced the mRNA expression of α-SMA (0.68 to 0.74-fold) (Figure 6E), collagen I (0.39 to 0.74-fold) (Figure 6F), MMP-2 (0.63 to 0.72-fold) (Figure 6G) and MMP-9 (0.61 to 0.68-fold) (Figure 6H), compared to expression levels in the MCD diet only group.

### 2.8. Effect of SME on mRNA Expression of NASH-Related Specific Genes in HSCs Induced by TGF-β1

TGF-β1 plays a pivotal role in hepatic fibrogenesis through HSC activation and increased synthesis of α-SMA and various ECM-related factors such as collagen I, MMP-2 and MMP-9 [16,17,19]. 

**Figure 7:**
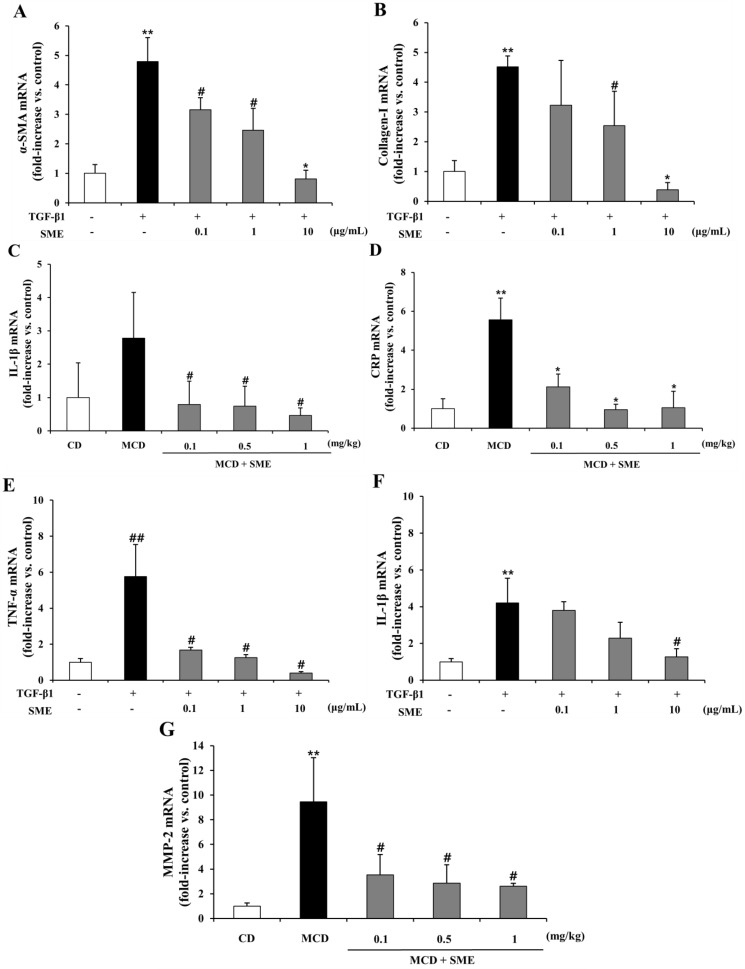
Effect of SME on mRNA expression of NASH-related specific genes in TGF-β1 induced LX-2 cells Comparison of LX-2 cells treated with TGF-β1 only (control) and SME plus TGF-β1 for 24 h. (**A**) α-SMA; (**B**) Collagen-I; (**C**) MMP-2; (**D**) MMP-9; (**E**) TNF-α; (**F**) IL-1β; (**G**) CRP. The mRNA expression level, normalized to GAPDH levels, is represented as fold induction of control and shown as mean ± SD (*n =* 9 per group), ** *p* < 0.01 *vs.* untreated; **^##^** *p* < 0.05 *vs.* untreated; * *p* < 0.01 TGF-β1 *vs.* SME treated; ^#^ *p* < 0.05 TGF-β1 *vs.* SME treated.

Therefore, TGF-β1was used as an HSC inducer in this study to investigate whether SME could inhibit HSC activation and progression. The pro-inflammatory cytokine, TNF-α, plays a major role in the progression from steatosis to NASH and also causes secretion of various other cytokines and chemokines [7]. Figure 7A–G shows that α-SMA, collagen I, MMP-2 and MMP-9 mRNA expression levels were increased by 4.79-, 4.51-, 3.47- and 4.65-fold, respectively, in the TGF-β1-treated cells compared to the normal group. Treatment with SME (0.1–10 μg/mL) reduced the mRNA expression of α-SMA (0.34 to 0.82-fold) (Figure 7A), collagen I (0.28 to 0.91-fold) (Figure 7B), MMP-2 (0.16 to 0.71-fold) (Figure 7C) and MMP-9 (0.68 to 0.79-fold) (Figure 7D) relative to the expression in cells treated only with TGF-β1. The mRNA expression of inflammatory cytokines and chemokines exhibited similar patterns. In the TGF-β1-treated cells TNF-α, IL-1β and CRP mRNA levels were increased by 5.75-, 4.20-, 6.14-fold, respectively, compared to the normal group. SME treatment (0.1–10 μg/mL) reduced the mRNA expression of TNF-α (0.71 to 0.93-fold) (Figure 7E), IL-1β (0.10 to 0.70-fold) (Figure 7F) and CRP (0.23 to 0.88-fold) (Figure 7G) relative to the expression in cells treated with TGF-β1 alone. 

### 2.9. Effect of SME on HSCs Induced by Oxidative Stress

**Figure 8:**
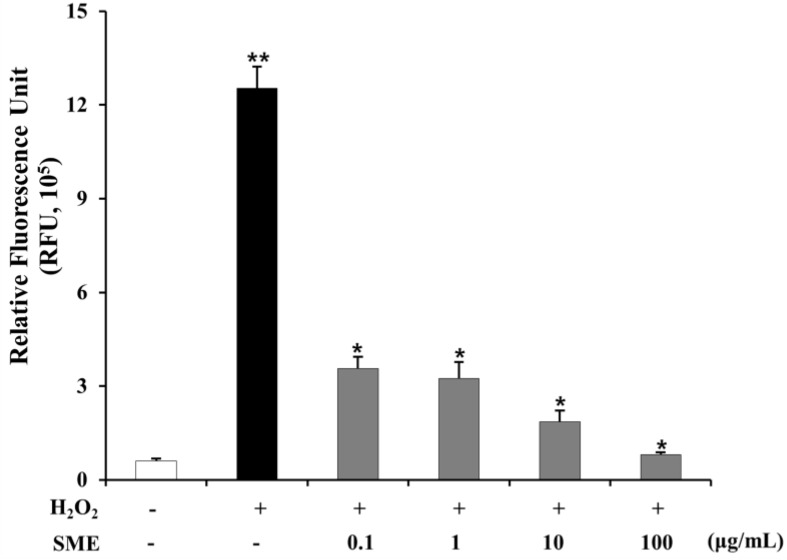
Effct of SME on oxidative stress in LX-2 cells LX-2 cells were either left untreated or pre-treated with SME (0.1-100 μg/mL) for 30 min and then left untreated or exposed to 600 μM hydrogen peroxide (H_2_O_2_) for 15 min. Intracellular generation of ROS was detected by measuring the conversion of 10 µM DCF-DA as described in experimental methods. Values are means ± SD (*n =* 9 per group), ** *p* < 0.01 *vs.* untreated; * *p* < 0.01 TGF-β1 *vs.* SME treated.

Oxidative stress plays an important role in the development of liver fibrosis, by activating different signaling molecules underlying NASH-related fibrogenesis [35]. ROS and TGF-β have a close relationship in hepatic fibrosis. ROS up-regulates TGF-β secretion in rat HSCs by latent expression of associated proteins. TGF-β also induces ROS production in mitochondria and microsomes and inhibits antioxidative activities in rat hepatocytes [36,37,38]. In this study, SME attenuated intracellular ROS generation in HSC. Figure 8 shows that intracellular ROS production of LX-2 cells treated with H_2_O_2_ was 20.71-fold higher than that of control cells (untreated) (*p* < 0.01). Co-treatment with SME (0.1–100 μg/mL) reduced ROS production in LX-2 cells (0.72 to 0.94-fold, *p* < 0.01) compared to cells treated with H_2_O_2_ alone.

## 3. Experimental

### 3.1. Preparation of Standardized Extract of Salvia Miltiorrhiza

Three batches (No. 20110801-20110803) of standardized *Salvia miltiorrhiza* extract (SME) containing 40% tanshinone IIA were manufactured and verified by Liwah Pharmaceutical Co., Ltd. (Ningbo, China). Briefly, the dried root of *Salvia miltiorrhiza* (Shandong, China/batch No. 1103004, Liwah) was shattered and boiled in 70% ethanol for 5 h. The extract was then filtered, vacuum dried, and recrystallized with ethyl acetate for 24 h. Finally, the concentrated extract was filtered, vacuum dried, and packaged in a vacuum aluminum foil bag and stored at 4 °C until use. The extraction yield was approximately 0.2% (w/w). For *in vivo* and *in vitro* studies, the dried extract was dissolved in 50% Polyethyleneglycol (PEG, Sigma-Aldrich, St Louis, MO, USA)-400 solution and 0.01% Dimethyl sulfoxide (DMSO, Sigma) before use. Previous studies confirmed stability and homogeneity of the extract in 50% PEG-400 at all concentrations used. Dose formulations were prepared by suspension of SME in 50% PEG-400, at determined concentrations 1 day in advance of administration, followed by storage at 4 °C. The limits of analytical results for all dose formulations were within 10% of the theoretical concentrations.

### 3.2. Stability Analysis of Tanshinone IIA by HPLC

The tanshinone IIA content in the SME was analyzed according to methods previously established by Samil Pharmaceuticals. Powdered extract (100 mg) and standards (each 4 mg; 98.2% HPLC purity, LKT Laboratories, St Paul, MN, USA) were completely dissolved in methanol solution for 30 min using a sonicator bath and the solutions were filtered through a membrane filter (0.45 μm, Millipore). Concentrations were determined by a HPLC system (Agilent 1200 HPLC, Agilent Technologies, Santa Clara, CA, USA) with a flow rate of 0.8 mL/min. HPLC conditions for the quantification of tanshinone IIA were as follows: Xterra (Waters, Milford, MA, USA) RP18 column (4.6 mm × 250 mm, 5 μm), 65% acetonitrile elution solvent and 270 nm detection wavelength. The injection volume was 10 μL for both standard solutions and sample. Peaks were identified by comparison with the retention time of the standards. For the accelerated, stressed and long-term stability test, SMEs packaged in low-density polyethylene (LDPE) bottles were stored at 40 °C/75%, 60 °C/75% and 25 °C/60% relative humidity (RH) for 12 weeks, respectively, and tanshinone IIA content was analyzed by the HPLC method as above. 

### 3.3. Animals and Experimental Protocol

Male C57BL/6J mice weighing 20–25 g were obtained from Orient Bio Inc. (Seongnam, Korea). All animals, 6 weeks of age at the beginning of the study, were housed in a controlled temperature of 20 ± 3 °C, 45%–60% RH and 12 h light/dark cycles. Mice had access to food and water ad libitum and were weighed at weekly intervals throughout the experiment. Animals were randomly divided into normal diet group (two groups of seven) or MCD diet group (eight groups of seven). Mice were fed either a methionine-choline deficient diet (MCD; MP Biomedicals, Solon, OH, USA) or control (standard chow) diet and tap water ad libitum for up to 8 weeks. All animals were acclimated to housing for 7 days prior to the first day of dosing. After 8 weeks of the MCD diet to induce the model of NASH, 70 animals were assigned to each treatment group (Table 2). At 9 weeks, SME in 50% PEG-400 (0.1, 0.5 and 1 mg/kg body weight, 0.01 mL/kg) was administered orally to MCD diet groups and an equal volume of vehicle (PEG-400) was administered orally to the control and normal group animals every other day for 4 and 6 weeks. Solutions were made fresh before each administration. Seven animals in each of the five experimental groups were euthanized at 4 and 6 weeks by exsanguination under anesthesia. At the end of the experiment, livers were rapidly dissected out, snap-frozen in liquid nitrogen and stored at −70 °C for evaluation of mRNA expression. A portion of the liver was fixed in 10% neutral formalin for histological evaluation. All experimental procedures were approved by the Institutional Animal Care and Use Committees (IACUC) of the Asan Institute for Life Science (ALIS).

### 3.4. Liver Histopathology

Liver specimens were fixed with 10% neutral formalin, dehydrated and embedded in paraffin. Tissue sections of 4 μm were stained by H&E and Sirius Red. Histological changes were observed with a microscope (Olympus BX53, Tokyo, Japan). 

**Table 2:** Experimental Design: after supplying MCD diet for 8 weeks to induce NASH model, seventy animals were assigned to an each treatment group.

Diet	Test Article	Group	Test Article Administration Period (weeks)	Dose Level	Number of Animals (male)
				(mg/kg)	
Normal Diet	Normal	1. Normal	4	0	7
		2. Normal	6		7
MCD Diet	Control	3. Control	4	0	7
		4. Control	6		7
	Low dose	5. MCD+Low dose	4	0.1	7
		6. MCD+Low dose	6		7
	Mid dose	7. MCD+Mid dose	4	0.5	7
		8. MCD+Mid dose	6		7
	High Dose	9. MCD+High dose	4	1	7
		10. MCD+High dose	6		7

#### 3.4.1. NASH (NAFLD Activity Score) Evaluation

Tissue sections of liver were stained by H&E. All H&E liver tissues were evaluated in a blinded manner by pathologists for steatosis, ballooning, and inflammation, according to the scoring method of Kleiner *et al*. [39] as follows: steatosis grade (0–3; 0:<5%, 1:5%–33%, 2: 33%–66%, and 3:>66%), lobular inflammation (0–3; 0:No foci, 1:<2 foci/200×, 2:2–4 foci/200×, and 3:>4 foci/200×), hepatocyte ballooning (0–2; 0:None, 1:few balloon cells, and 2:many cells/prominent ballooning). 

#### 3.4.2. Fibrosis Evaluation and Measurement of Collagen Distribution

The Sirius Red stain, which stains collagen a dark pink color, was utilized for liver fibrosis evaluation. A computer scanning program (Panoramic viewer, 3DHistech, Budapest, Hungary) was used to measure collagen area and whole liver area in each section. The ratio of collagen area to whole liver area was calculated after ruling out any artifacts. The measured color range was hue: 130–245, saturation: 52–213, and value: 220–248.

### 3.5. Cell Culture & Reagents

The human cell line of HSCs (LX-2 cells were kindly supplied by Dr. S.G. Kim (College of Pharmacy, Seoul National University)) were cultured in Dulbecco’s modified Eagle’s medium (DMEM) containing 10% fetal bovine serum (FBS), penicillin (100 U/mL), and streptomycin (100 μg/mL) at 37 °C with 5% CO_2_. When the cultures reached confluence, cells were trypsinized and passaged. Subsequent passages were performed every 5–7 days. LX-2 cells were seeded in 6-well culture plates (3 × 10^5^ cells per well). Then, 24 h later, medium was changed to DMEM with 2% FBS, penicillin and streptomycin for 24 h to achieve synchronization. After that, cells were respectively treated with vehicle (control), TGF-β1 (5 ng/mL) and TGF- β1 plus SME (0.1–10 μg/mL) for 24 h, and harvested to investigate the expression of target genes. DMEM, FBS, penicillin and streptomycin were purchased from Gibco (Carlsbad, CA, USA). Recombinant human transforming growth factor β1 (TGF-β1) was obtained from R&D systems (Minneapolis, MN, USA). 

### 3.6. Preparation of Liver Lysate

Frozen livers tissues were lysed using T-PER tissue protein extraction reagent (Thermo Scientific, Rockford, IL, USA) containing Halt protease inhibitor mixture (Thermo Scientific) and samples were homogenized on ice using the Tissue-ruptor (Qiagen, Valencia, CA, USA). Tissue debris was removed by centrifugation for 5 min at 10,000 rpm. Supernatant was collected, and total protein concentration was determined by the bicinchoninic acid (BCA) assay (Pierce, Rockford, IL, USA).

### 3.7. RNA Isolation and Quantitative PCR

Total RNA from liver tissue and LX-2 cells were separately isolated according to the protocol of the RNeasy Plus Mini Kit (Qiagen). RNA purity was confirmed by a 260/280 nm absorbance ratio greater than 1.8. First strand cDNA synthesis was performed by reverse transcription with 1 μg total RNA using the Transcriptor First Strand cDNA Synthesis Kit (Roche Diagnostics, Mannheim, Germany). The synthesized cDNA was used as a template to estimate the quantity of gene transcription by real-time PCR. Specific primers (Bioneer, Daejeon, KOREA) and Universal Probe Library (UPL, Roche Diagnostics) probes were designed and auto-made by the National Center for Biotechnology Information (NCBI) gene related database and Roche Diagnostics. The amplification reactions were performed on a Roche light cycler 480 instrument (Roche Diagnostics) using Lightcycler^®^ 480 probes master (Roche Diagnostics) and specific primers (Table 3). The amplification conditions were as follows: 95 °C (10 s), 60 °C (30 s), 72 °C (1 s) for 45 cycles. The primers and probes for specific genes or glyceraldehyde-3-phosphate dehydrogenase (GAPDH) are listed in Table 1. The relative quantitation was calculated as 2^−∆Ct^ (∆Ct = Ct of the target gene minus Ct of GAPDH).

**Table 3:** List of the sets of probes and primers (Roche Diagnostics) for specific genes, used in quantitative real-time PCR.

Target Gene	Primer (Forward/Reverse)		Reference	
***Human***				
α-SMA	**F:** ctg ttc cag cca tcc ttc at	**R:** tca tga tgc tgt tgt agg tgg t		ENSG00000107796
MMP-2	**F:** ccc caa aac gga caa aga g	**R:** ctt cag cac aaa acg gtt gc		ENSG00000087245
MMP-9	**F:** tct tcc ctg gag acc tga ga	**R:** gag tgt aac cat agc ggt aca gg		ENSG00000100985
Collagen I	**F:** ggg att ccc tgg acc taa ag	**R:** gga aca cct cgc tct cca		ENSG00000108821
TNF-α	**F:** cag cct ctt ctc ctt cct gat	**R:** gcc aga ggg ctg att aga ga		ENSG00000232810
CRP	**F:** cca gct gtg ggt cct gaa	**R:** cac agc ccc aca agg ttc		ENSG00000132693
IL-1β	**F:** agc tga tgg ccc taa aca ga	**R:** gtc gga gat tcg tag ctg ga		ENSG00000125538
***Mouse***				
α-SMA	**F:** ctc tct tcc agc cat ctt tca t	**R:** tat agg tgg ttt cgt gga tgc		ENSMUSG00000035783
MMP-2	**F:** aac ttt gag aag gat ggc aag t	**R:** tgc cac cca tgg taa aca a		ENSMUSG00000031740
MMP-9	**F:** acg aca tag acg gca tcc a	**R:** gct gtg gtt cag ttg tgg tg		ENSMUSG00000017737
Collagen I	**F:** ctg gtc ctg ctg gct ttg	**R:** acc acg atc gcc att ctt		ENSMUSG00000001506
TNF-α	**F:** cca gac cct cac act caca a	**R:** ttg aga tcc atg ccg ttg		ENSMUSG00000024401
CRP	**F:** ctc gga ctt ttg gtc atg aag	**R:** aaa ggt gtt cag tgg ctt ctt t		ENSMUSG00000037942
IL-1β	**F:** agt tga cgg acc cca aaa g	**R:** ttt gaa gct gga tgc tct cat		ENSMUSG00000027398
TGF-β1	**F:** tgg agc aac atg tgg aac tc	**R:** gtc agc agc cgg tta cca		ENSMUSG00000002603

### 3.8. Enzyme-Linked Immunosorbent Assay (ELISA)

The production of TNF-α and type I collagen in mice liver tissues was measured using commercial mouse ELISA kits (TNF-α; R&D Systems, Minneapolis, MN, USA/Type I collagen; Chondrex, Redmond, WA, USA) according to the manufacturer’s instructions. Protein samples were prepared with 50 mg liver tissue using a method described previously. The result was calculated as protein concentration in 100 μg liver lysates.

### 3.9. Intracellular ROS Activity

Reactive oxygen species (ROS) activity was quantified using an Oxiselect intracellular ROS assay kit (Cell Biolabs, San Diego, CA, USA). Briefly, the LX-2 cells were seeded in 96-well culture plates (2 × 10^4^ cells per well). After 24 h of incubation in DMEM containing 2% FBS, cells were pre-incubated for 30 min with SME (0.1–100 μg/mL) and then left untreated or exposed to 600 μM hydrogen peroxide (H_2_O_2_) for 15 min. Intracellular ROS level was detected by measuring the conversion of 10 µM 2',7'-dichlorofluorescein diacetate (DCF-DA). Fluorescence intensity was determined using VICTOR X2 Multilabel Plate Reader (Perkin-Elmer, Heidelberg, Germany) at 485 nm excitation and 535 nm emission. 

### 3.10. Statistical Analysis

All statistical analyses were performed with SPSS software version 12.0 (SPSS Inc., Chicago, IL, USA). Values were expressed as means ± SD of independent experiments. Statistical significance for differences between the groups were performed using the unpaired Student’s *t*-test or one-way ANOVA. Data with a *p* value less than 0.05 were considered statistically significant.

## 4. Conclusions

We have shown that standardized SME administration limits NASH development and its related cytopathic progression in mice with experimental steatohepatitis and human HSC. SME achieves its effects by controlling the expression of inflammatory and fibrogenic cytokines. Importantly, our results suggest that SME may improve hepatic function in NASH patients, thus SME may be a potential herbal medicine for the treatment of NAFLD.

## Supplementary Materials

Supplementary materials can be accessed at: http://www.mdpi.com/1420-3049/19/6/8189/s1.
