# Supplementary material for: Standardized Salvia miltiorrhiza Extract Suppresses Hepatic Stellate Cell Activation and Attenuates Steatohepatitis Induced by a Methionine-Choline Deficient Diet in Mice
Source: Molecules. 2014 Jun 17;19(6):8189–211. doi: 10.3390/molecules19068189 (PMC6271030; doi:10.3390/molecules19068189)

Supplementary Materials

Figure 1. Survival curves of animals used in MCD diet-induced NASH model. After 8 weeks of the MCD diet to induce the model of NASH, animals were treated with MCD diet plus SME (0.1, 0.5 and 1 mg/kg body weight, 0.01 mL/kg) **(A)** 4 weeks and **(B)** 6 weeks period.

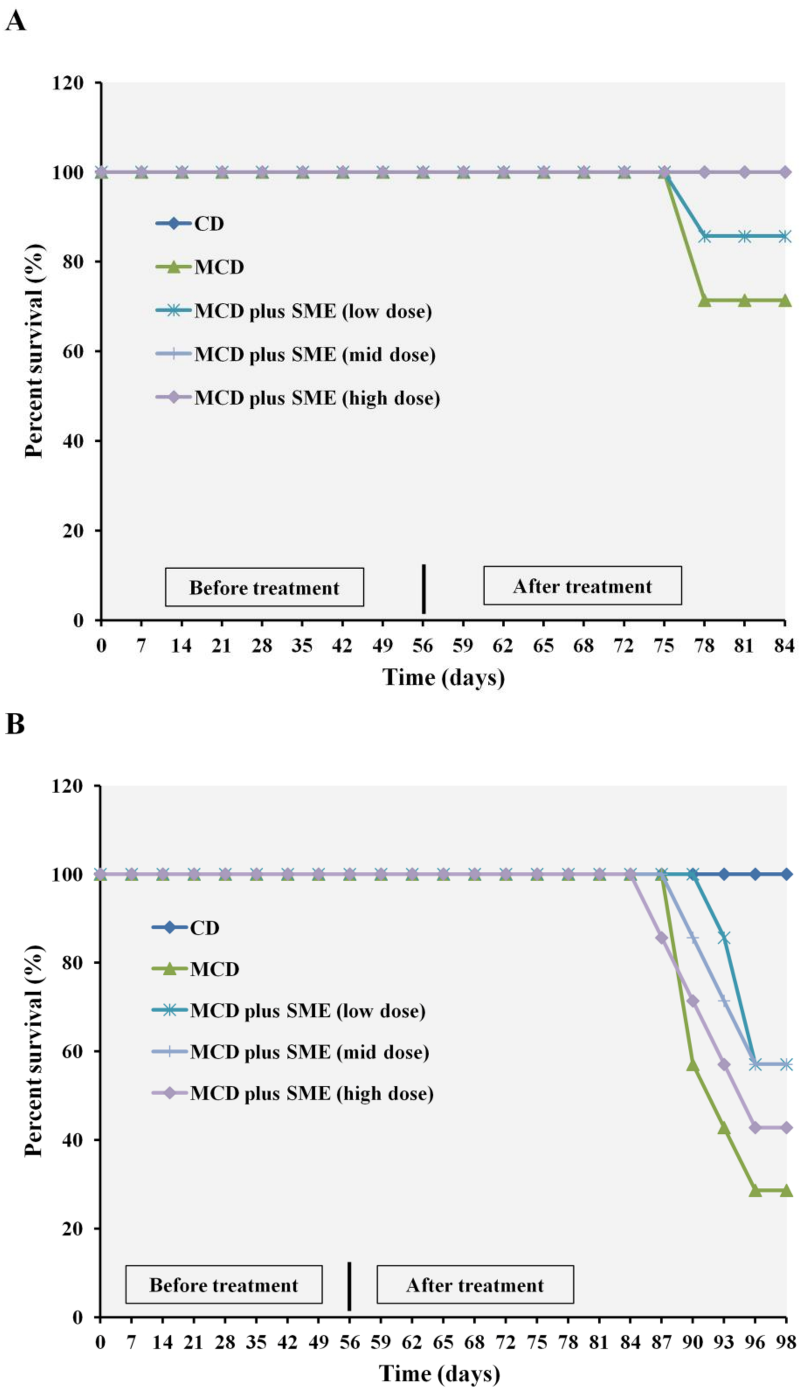

Figure 2. Stability test of cryptotanshinone, tanshinone I and tanshinone IIA SME packaged in low-density polyethylene (LDPE) bottles were stored at 40 ℃/75% (Inter mediate), 60 ℃/75% (Accelerated) and 25 ℃/60% (Long term) relative humidity (RH) for 12 weeks, respectively. **(A)** Cryptotanshinone, **(B)** Tanshinone I, **(C)** Tanshinone IIA

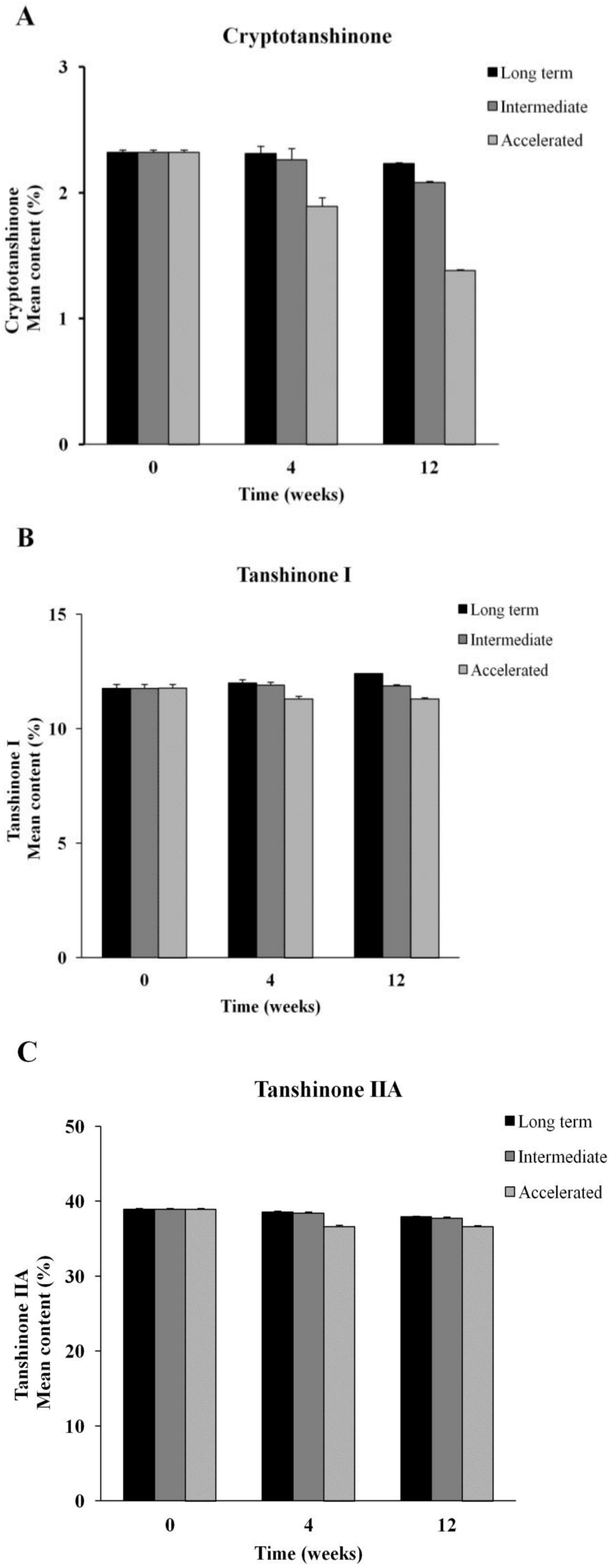

Supplement: Supplementary file 1 [file molecules-19-08189-s001.pdf]
